# STAT3/miR-15a-5p/CX3CL1 Loop Regulates Proliferation and Migration of Vascular Endothelial Cells in Atherosclerosis

**DOI:** 10.7150/ijms.49460

**Published:** 2021-01-01

**Authors:** Hui Li, Hui-Min Zhang, Li-Juan Fan, Han-Han Li, Zi-Tan Peng, Jia-Peng Li, Xiao-Yu Zhang, Yuan Xiang, Chao-Jiang Gu, Xing-Hua Liao, Li Wang, Tong-Cun Zhang

**Affiliations:** 1 College of Life Sciences and Health, School of Resource and Environmental Engineering, Wuhan University of Science and Technology, Hubei, 430081, P.R.China.; 2 College of Life Sciences and Health, Wuhan University of Science and Technology, Hubei, 430081, P.R.China.; 3 Tianyou Hospital Affiliated To Wuhan University of Science and Technology, Hubei, 430000, P.R.China.; 4 Huangshi Central Hospital, Hubei, 435000, P.R.China.; 5 School of Resource and Environmental Engineering, Wuhan University of Science and Technology, Hubei, 430081, P.R.China.; 6 Key Laboratory of Industrial Fermentation Microbiology, Ministry of Education and Tianjin, College of Biotechnology, Tianjin University of Science and Technology, Tianjin, 300457, P.R.China.

**Keywords:** STAT3, MicroRNA-15a, CX3CL1, Endothelial cells, Atherosclerosis

## Abstract

Endothelial cell proliferation disorder caused by vascular injury seems to be one of the causes of atherosclerosis, which is the pathological basis of coronary heart disease. The role of STAT3 in the regulation of microRNAs and endothelial dysfunction in atherosclerosis is unclear. STAT3 can be activated by cytokine IL-6 and up regulate the expression of CX3CL1. In addition, microRNA-15a-5p (miR-15a-5p) inhibited the transcription of CX3CL1, the proliferation of vascular endothelial cells and the proliferation of STAT3 regulated vascular endothelial cells. STAT3 positively regulates the expression of CX3CL1, and then down-regulates the inhibition of CX3CL1 by over-expression of miR-15a-5p, thus forming an elimination feedback loop to control the proliferation of HUVECs and affect the progression of atherosclerosis. In conclusion, miR-15a-5p may be the therapeutic target of the pathological basis of coronary atherosclerosis.

## Introduction

Although the morbid etiopathogenesis is not entirely decipherable, atherosclerosis is known as a complex and chronic inflammatory disease 1,2. Endothelial dysfunction occurs at the beginning of atherosclerosis and leads to hardening and thickening of the arterial wall and plaque formation 3. These plaques include immune cells, mesenchymal cells, lipids and extracellular matrix. The source of mesenchymal cells in atherosclerotic plaques has been under scrutiny for many years. Current endothelial lineage tracking studies show that endothelium is the source of mesenchymal cells associated with plaques. Chemokine (C-X3-C motif) unique ligand 1 (CX3CL1) exists in neurons, intestinal epithelium, activated vascular endothelial cells and other cell types, and participates in neuroinflammatory response 4. Partial studies have showed that CX3CL1 and its receptor CX3C receptor1 (CX3CR1) play a key player in vascular inflammation and injury. The levels of MIP-1β, TNF-α and CX3CL1 reflect the levels of several atherogenic cytokines in plaque tissues, and are potential markers for identifying patients with high-risk plaques 5. Both CX3CL1 and its receptor CX3CR1 can be expressed on endothelial cells. The CX3CL1/CX3CR1 axis may induce the proliferation, migration and formation of endothelial cells in vitro and stimulate angiogenesis in vivo 6. The level of CX3CLl in clinical specimens is closely related to various heart failures, such as carotid stenosis 7, unstable angina 8,9, systolic heart failure, abdominal aortic aneurysm 10, and chronic obstructive pulmonary disease-related cardiovascular diseases 11. CX3CL1 activates the pro-inflammatory pathway mediated by the transcription factor NF-κB, which is an early response of microglia 12.

Endothelial homeostasis dysfunction induces the differential expression of chemokine molecules, and at the same time, chemokine molecules chemoattract leukocytes are the beginning of the inflammatory process 13. The JAK/STAT3 pathway is the classical pathway of STAT3 activation. Recombinant human interleukin 6 (IL-6) is most closely related to the activation of STAT3 in extracellular cytokine signaling. It can promote the continuous activation of various cytokines and growth factors 14,15, and is accompanied by persistent chronic inflammation^16^. Therefore, it is of considerable significance to study the interaction between STAT3 and multiple cytokines, include CX3CL1, E-selectin, and other potential adhesion molecules 17,18.

The microRNAs (miRNAs) mostly been evolutionarily conserved and have a substantial contribution to cardiovascular physiology and pathology. The anomalous expression of miR-15a-5p in specimens has involved in acute coronary syndrome and inflammation 19, might even to atherosclerosis and cardiovascular diseases. In addition, it is generally believed that miRNAs can regulate the function of cell signaling pathways by binding to the pairing sites on the 3'untranslated region (3'-UTR) of potential target mRNAs, which belongs to post-transcriptional inhibition and regulation of mRNA expression 20. However, the role of miR-15a-5p in the proliferation and migration of HUVEC cells (ECs) in atherosclerosis remains unclear.

According to the results of the TargetScan website, CX3CL1 has a sole potentiality of the binding site for miR-15a-5p, which could potentially recognize the target sequence on CX3CL1. It may have the characteristics of post-transcriptional regulation to regulate the synthesis of CX3CL1 protein. Almost all STAT families can recognize specific sequences (TGCN_2-4_GAA), which is a typical small DNA palindrome of the interferon gamma activation site (GAS). Our present research was to investigate the physiological function of miR-15a-5p in AS pathogenesis. In particular, the molecular mechanism of STAT3/miR-15a-5p-CX3CL1 axis in the proliferation and migration of endothelial cells was proposed. It is of considerable significance to reveal how the destruction of physiological balance changes to the pathological state.

## Methods

### Animals and Cell lines

8-week-old male mice, ApoE^-/-^ mice (C57BL/6) and C57BL/6 mice (Purchased from Model Animal Research Center of Nanjing University (China)), were fed a specially configured high-fat Feed (40% fat (20%w), 1.25% cholesterol; Trophic Animal Feed High-tech Co., Nanjing, China)^21^ for eight weeks. The feeding environment for rats must have the following three points: keep a room at 22°C, drink pure water freely, and cycle day and night. Mice thoracotomies were performed under a sterile environment with the backing of an operating stereomicroscope. The use of human umbilical vein fusion cell line HUVEC-EA.hy926 (HUVECs) can well represent endothelial cells and reflect their characteristics. HUVEC-EA.hy926 (ATCC) must contain: high glucose Dulbecco's modified Eagle's medium (DMEM)(GIBCO) (fetal bovine serum with a final concentrate of 10% and 10U/ml penicillin-streptomycin), 37℃ and 5% CO_2_ incubator. Cells at 60% confluence cultured as routine in DMEM were activated by experimental incubation on different time and different density.

### Structure of luciferase plasmids and reporter assay

The expression plasmid pcDNA3.1-STAT3 encoding human STAT3 was constructed in our laboratory and described previously 22. The promoter areas of miR-15a-5p (-300 to +30) and CX3CL1 (-992 to +19) were augmented by conventional PCR replaced by cloning into pGL3 luciferase reporter vector. The primers used to generate target promoter-Luc plasmids were as follows: hsa-miR-15a-5p F 5'-AAAAGGTACCAGCATTTAGTTGTATTGCCCTGTTA-3' and R 5'-AAAAGAGCTCACCATTATGTGCTGCTACTTTACTC 3', CX3CL1 5'-TACCGAGCTCTTACGCGTGCTAGCAGACTGTGTTCTAATGTGCT-3' 5'-CCAACAGTACCGGAATGCCAAGCTTATCTGTGGCTTTTTATAATG-3'. The vector pGL-3 (Promega) was devoted as a control. In addition, the primers used to create CX3CL1-3'UTR-Luc was as follows: CX3CL1-3'UTR: F 5'-AAAAGGTACCAGCATTTAGTTGTATTGCCCTGTTA-3' and R 5'-AAAAGAGCTCACCATTATGTGCTGCTACTTTACTC-3', the vector pmirGLO (Promega) was used as a control.

The prophesied target sites were mutated by site-directed Mut Express II Fast Mutagenesis Kit V2 (Vazyme, China). Wildtype (WT) or mutated (MUT) luciferase plasmids and pcDNA 3.1-STAT3 or microRNAs were co-transfected into cells. Luciferase activity was detected after 24 hours of co-culture. Repeat each experiment three times to reduce system error.

### Antibodies, western Blotting

Antibodies: rabbit STAT3 (ab92552) (mAbs, Abcam), rabbit Phospho-STAT3 (Ser727) (AF3294) (PcAb, Affbiotech), rabbit CX3CL1 (ab25088) (PcAb, Abcam) and mouse GAPDH (sc-51907) (mAbs, Santa Cruz). Secondary antibodies for western blot: DyeTM-800 anti-rabbit antibodies and IR DyeTM-680 anti-mouse antibodies(Li-COR). IL-6 was obtained from Peprotech Inc (Rocky Hill, NJ). The specific steps of the western blotting experiment are as described previously^22^,^23^. The membranes were visualized by GelDoc XR+ Imaging System (Bio-Rad), examination of the image was examined using the ImageJ software.

### siRNA gene silencing and microRNA transient transfection

The online prediction programs of microRNAs, including Targetscan (http://www. targetscan.org), PicTar (https:// pictar.mdc-berlin.de/) and miRanda (https: //www. microrna.org/microrna/ home. do), are used to predict microRNAs associated with CX3CL1. All microRNAs, siRNA and their corresponding control (NC) for RNA transfection were designed and purchased from Guangzhou RIBOBIO Co. The transfection efficiency was analyzed and confirmed by additional biological tests. The suitable transfection reagent is Lipofectamine® 2000 Reagen (Thermo Fisher) according to the appropriate experimental groups and manufacturer's protocol. A total of 1×10^6^ cells mixed with 100 pmol of siRNA in 100 µL of 37℃ DMEM. Subsequent incubation for 6 h, the media was displaced with regular culture medium for 48 h. The effectiveness of transfection was assessed by siRNA-cy3-control expression.

### Immunofluorescence

HUVECs processed on glass cap slides were tested for cellular immunofluorescence to observe the expression of intracellular protein, and the procedure of cell immunofluorescence test was described previously. Laser scanning confocal microscopy (Olympus) was used to observe the expression of FITC-goat anti-rabbit (ZFGB-BIO) (green) prominent protein, and 4',6-diamidino-2-phenylindole (DAPI) staining (blue) highlighted the whole nucleus.

### Quantitative real-time RT-PCR (qRT-PCR) and ChIP assays

qRT-PCR analysis and the ChIP experiment was done as described previously^24^. Glyceraldehyde-3-phosphate dehydrogenase (GAPDH) expression level was used as the relative standardization of data processing. ChIP analysis was performed using a commercially available kit-Enzymatic Chromatin IP (Magnetic Beads) (Cell Signaling Technology). A nonspecific IgG antibody was served as a negative control in our studies.

### Serum level of VEGF-A and CX3CL1 detected by ELISA

To verify our hypothesis, we utilized polyclonal antibodies (Abs) against VEGF-A (MMV00, R&D Systems) and CX3CL1 (MCX310, R&D Systems) by an extremely sensitive and specific enzyme-linked immunosorbent assay (ELISA), able to detect and quantify the level of inflammation in medium samples. The test sample was mouse root tip blood sample, centrifuged at 1200 g for 15 minutes to obtain supernatant. The ELISA experiment was performed as described previously 25.

### Ethynyl-2'-deoxyuridine (EdU)-labeling in cells proliferation and MTT assay

The EdU (5-Ethynyl-2'-deoxyuridine)-labeling procedure was previously reported 26,27. HUVECs cells on a 96-well plate as follows: siRNA-CX3CL1 (siCX3CL1) and control (si-NC), miR-15a-5p-mimic and its paired negative control (N.C.) by twenty-four hours pretreatment were treated 2 hours by EdU. DNA replication was determined using a Cell-LightTM EdU Kit (RIBOBIO, China), measuring numbers of positive cells labeled with nucleotide EdU and compound dyed with DAPI and mounted with Vectashield. Statistical analyses were performed using GraphPad Prism 6 software to evaluate the cell proliferation ability. Water-soluble MTT (3 - (4,5-dimethylthiazol-2-yl) - 2,5-diphenyltetrazolium bromide) was used to detect cell activity at 570nm.

### Migration assays

Cell migration ability was analyzed by cell scratch test and transwell test. Photographs of EA.hy926 cells were sensed at 0 h and 24 h after scratched of each experiment, and gap closure was determined on phase-contrast images by using the Time Lapse. Cell motility was experimented in an 8‐μm‐pore polycarbonate membrane (Corning, China) by transwell test. The DMEM containing 10% FBS was added into the basement and the cells were cultured in the upper layer for 48 hours. The cells without migration function were erased from the top of the membrane and the remaining migrated cells were stained in Giemsa and analyzed. The cells were calculated under a microscope (Olympus Corporation, Japan) in five distinct fields in duplicate wells, in at least three independent experiments.

### Statistical analyses

The experimental results were analyzed by SPSS software 17.0 (SPSS Inc.). The significance of the difference between samples was determined by student's 2-tail t test or one-way ANOVA. The experimental results were statistically analyzed according to the means ± standard deviation (SD), and each independent experiment was repeated at least three times.

### Statement

The experiment was carried out in accordance with the guidelines of the ethics committee of Tianyou Hospital Affiliated to Wuhan University of science and technology. The anesthetic used in the experiment was ether. During euthanasia, the mice inhaled ether first, then cervical dislocation. All the purchased animals were raised and tested in the experimental animal center of Huangjiahu campus of Wuhan University of science and technology.

## Results

### CX3CL1 induced proliferation and migration in HUVECs

**Figure 1:**
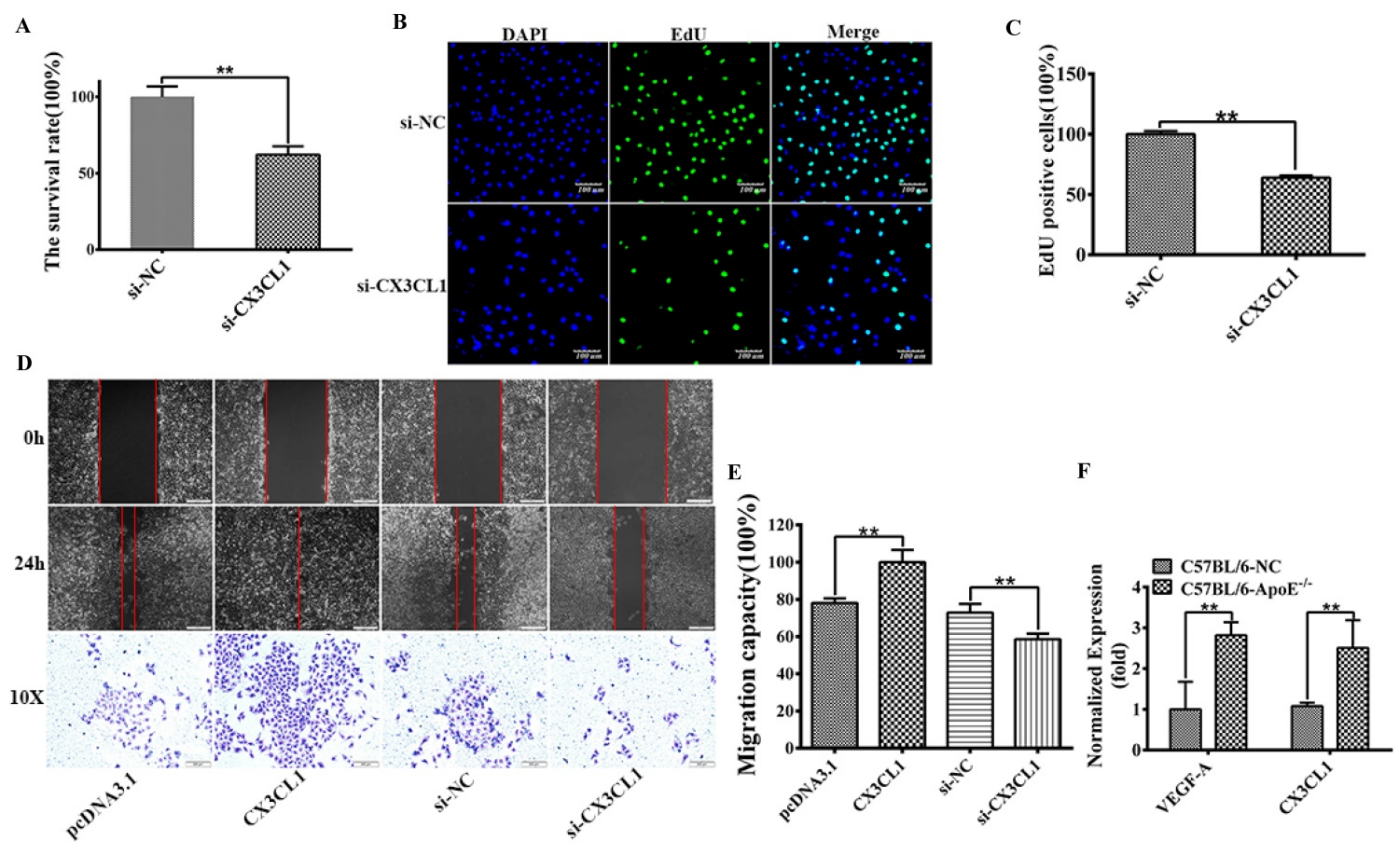
** CX3CL1 induced proliferation and promoted migration in HUVECs.** HUVECs after serum starvation (DMEM+0.5% FBS) for 48 hours were transfected with si-control (si-NC) or si-CX3CL1 for 48 hours. 8-week-old male mice were fed a high fat-, high cholesterol diet (40% fat (20%w), 1.25% cholesterol for eight weeks. **A:** Cell proliferation was measured by counting cell numbers as described in “Experimental Procedures.” n=3. Data are expressed as mean±S.D (**P< 0.01). **B and C:** Cell proliferation was measured by EdU assay. n=3. Data are expressed as mean±S.D (**P< 0.01). **D and E:** The effect of overexpressed or knocked down CX3CL1 on HUVECs migration of was detected by Wound-healing assay. (**P< 0.01). The transwell assay was also used to detect the migration-stimulating effects of overexpressed or knocked down CX3CL1 on HUVECs. The number of cells migrated to the lower side of the transwell chambers was counted and photographed in five fields (the upper, the lower, the left, the right, and the middle) of three independent experiments, and the migration capacity was calculated by SPSS statistical soft. (**P< 0.01). **F.** The effect of atherosclerosis on the expression of VEGFA and CX3CL1 was examined by ELISA assay in ApoE^-/-^ mice. (**P< 0.01)

To uncovering the function of CX3CL1 in HUVECs proliferation, MTT experiment and EdU positive cell analysis were performed to detect the cell survival rate and proliferation after transfected with siCX3CL1 and si-NC in EA.hy926 cells. The results showed that siCX3CL1 decreased the relative survival rate by about 40% (Figure 1A, **P< 0.01), reduced the normalized number of EdU positive cells (Figure 1 B and C, **P< 0.01). We also assessed whether CX3CL1 could promote the collective migration of HUVECs. The results showed that the ability of invasion increased when transfected with CX3CL1 while decreased when transfected with si‐CX3CL1 in ECs compared with the corresponding negative control group (Figure 1D and E, **P< 0.01). To examine the effect of the CX3CL1 and inflammation level in atherosclerosis model, seven-week-old apoE^-/-^ mice and C57BL/6 control mice were fed high-fat diet until they reached 16 weeks of age. AS shown in Figure 1F, up-regulated CX3CL1 exposed in serum samples secreted significantly higher levels of inflammatory factors VEGF-A compared with controls (Figure 1F, **P< 0.01).

### CX3CL1 expression in response to activation of the IL-6 / STAT3 signaling pathway

**Figure 2:**
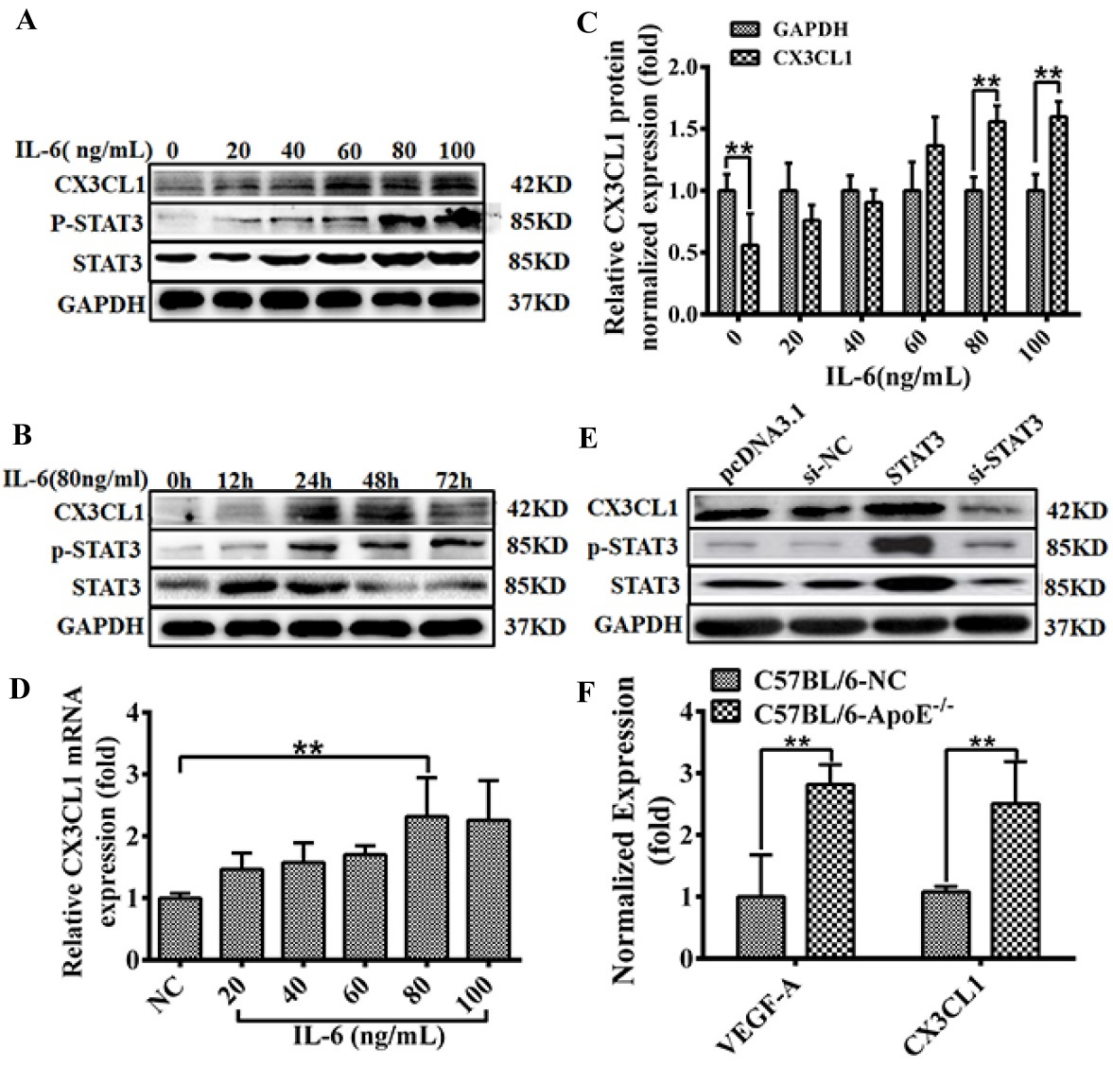
** CX3CL1 expression activated in HUVECs by STAT3 signaling pathway. A-D:** The effect of treated with IL-6 on CX3CL1 expression in HUVECs was detected by western blot analysis and qRT-PCR. Cells were stimulated by IL-6 at various concentrations (0, 10, 20, 40 60 and 80, 100 ng/mL) for 24 hours or at different times (0, 6, 12, 24, and 48h) at a concentration of 80ng/mL. All experiments were repeated three times in duplicate, and the semi-quantitative analysis was calculated by SPSS statistical soft. (**P< 0.01). **E and F:** The effect of treated with overexpression STAT3 or knocked down STAT3 on the expression of CX3CL1 was examined by western blot analysis and qRT-PCR in HUVECs. All experiments were repeated three times in duplicate, and the semi-quantitative analysis was calculated by SPSS statistical soft. (**P< 0.01)

STAT3 has been perfected as a new activator of CX3CL1 in vascular endothelial cells. In order to accurately explore the expression regulation mode of CX3CL1, we used gradient concentrations (0, 10, 20, 40, 60 and 80, 100 ng/mL) of IL-6 or different times (0, 6, 12, 24, and 48 h) to incubate the cells and then detect the expression of related genes. The results indicate that IL-6 stimulates the STAT3 pathway in a time- and dose-dependent manner (Figure 2A-D, ** *P* <0.01). In addition, pretreatment with IL-6 (80 ng/mL, 30 minutes) activated CX3CL1 expression (Figure 2B), suggesting that CX3CL1 expression was in response to IL-6 / STAT3 stimulation in HUVECs. After transfection of STAT3 or si-STAT3 into EA.hy926 cells for 24 hours, the expression of related genes was detected. The results showed that the overexpression of STAT3 could significantly increase the expression of CX3CL1, while knocking down STAT3 down-regulated the expression of CX3CL1 (Figure 2E and F, ** *P* <0.01). These results indicate that the expression of CX3CL1 is responsive to the stimulation of the IL-6 / STAT3 signaling pathway in an inflammatory state.

### MiR-15a-5p inhibits HUVECs proliferation and migration

**Figure 3:**
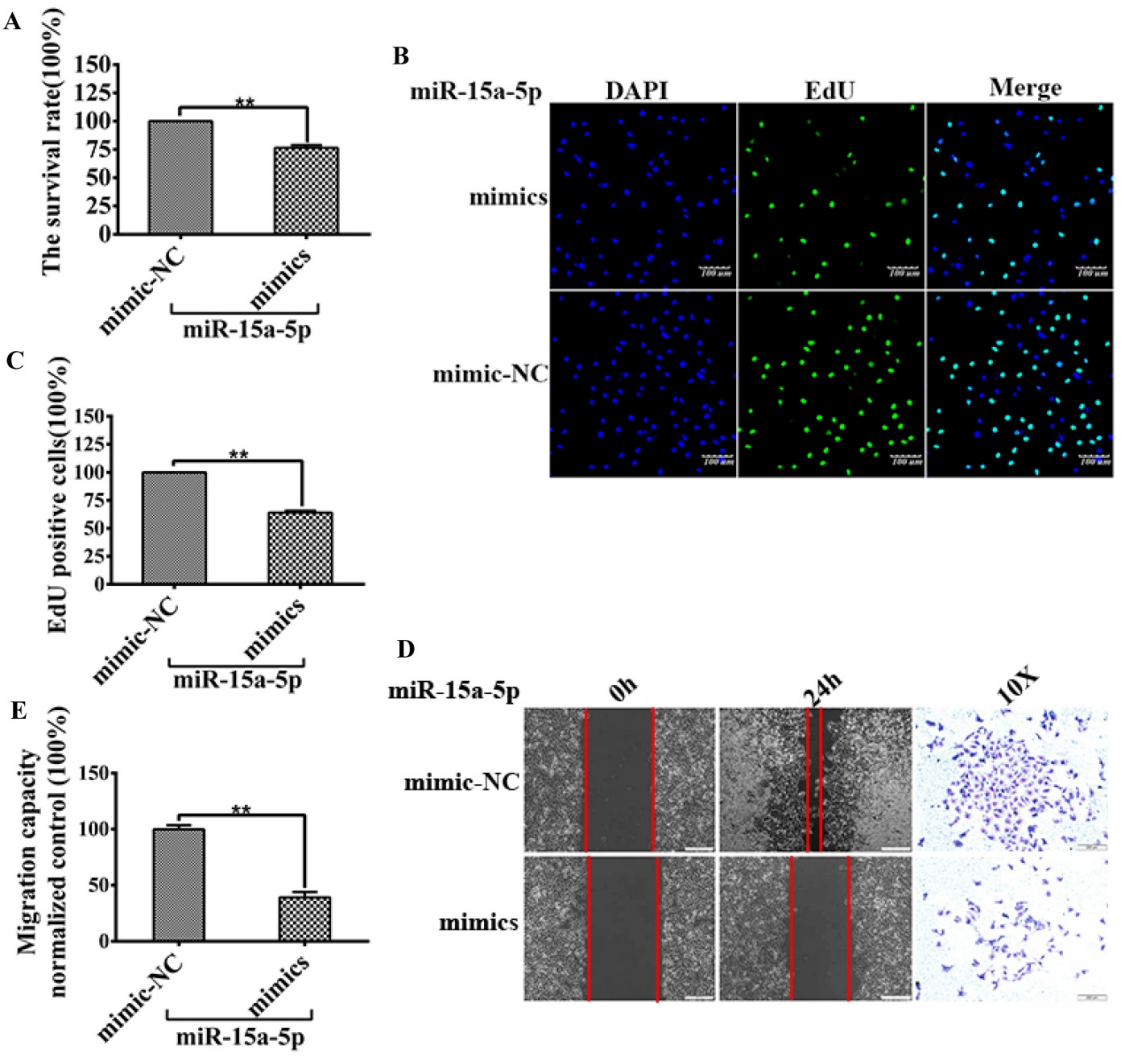
** MiR-15a-5p decreased CX3CL1 and inhibited HUVECs proliferation and migration.** HUVECs after serum starvation (DMEM+0.5% FBS) for 48 hours were transfected with miR-15a-5p (mimics) or respective negative control (NC) for 48 hours. **A:** Cell proliferation was measured by counting cell numbers as described in “Experimental Procedures.” n=3. Data are expressed as mean±S.D (**P<0.01). **B and C:** Cell proliferation was measured by EdU assay. n=3. Data are expressed as mean±S.D (**P<0.01). **D and E:** The effect of overexpressed miR-15a-5p (mimics) or respective negative control (NC) on HUVECs migration of was detected by Wound-healing assay. ( *P< 0.05, **P< 0.01). The transwell assay was also used to detect the migration-stimulating effects of overexpressed miR-15a-5p (mimics) or respective negative control (NC) on HUVECs. The number of cells migrated to the lower side of the transwell chambers was counted and photographed in five fields (the upper, the lower, the left, the right, and the middle) of three independent experiments, and the migration capacity was calculated by SPSS statistical soft.(**P< 0.01)

To explore the specific role of miR-15a-5p in CVD and its mechanism, miR-15a-5p mimics and mimic-NC and their corresponding controls were transfected into EA-hy926. MTT and EdU analysis was performed to detect HUVECs proliferation, the results showed that the relative survival rate of cells transfected with miR-15a-5p mimic was lower than these transfected with control (mimic-NC) (Figure 3A), and the number of EdU-positive cells was significantly lower than that of the control group (Figure 3B and C).

Wound healing and transwell analysis were performed to detect HUVECs migration. The results showed that the wound healing speed of miR-15a-5p mimic transfection group was faster than that of the control group, and the number of cells transferred to the lower side of the transwell chamber was also higher than that of the control group (Figures 3D and E).

### miR-15a-5p inhibits CX3CL1 protein expression by targeting CX3CL1 3'UTR

**Figure 4:**
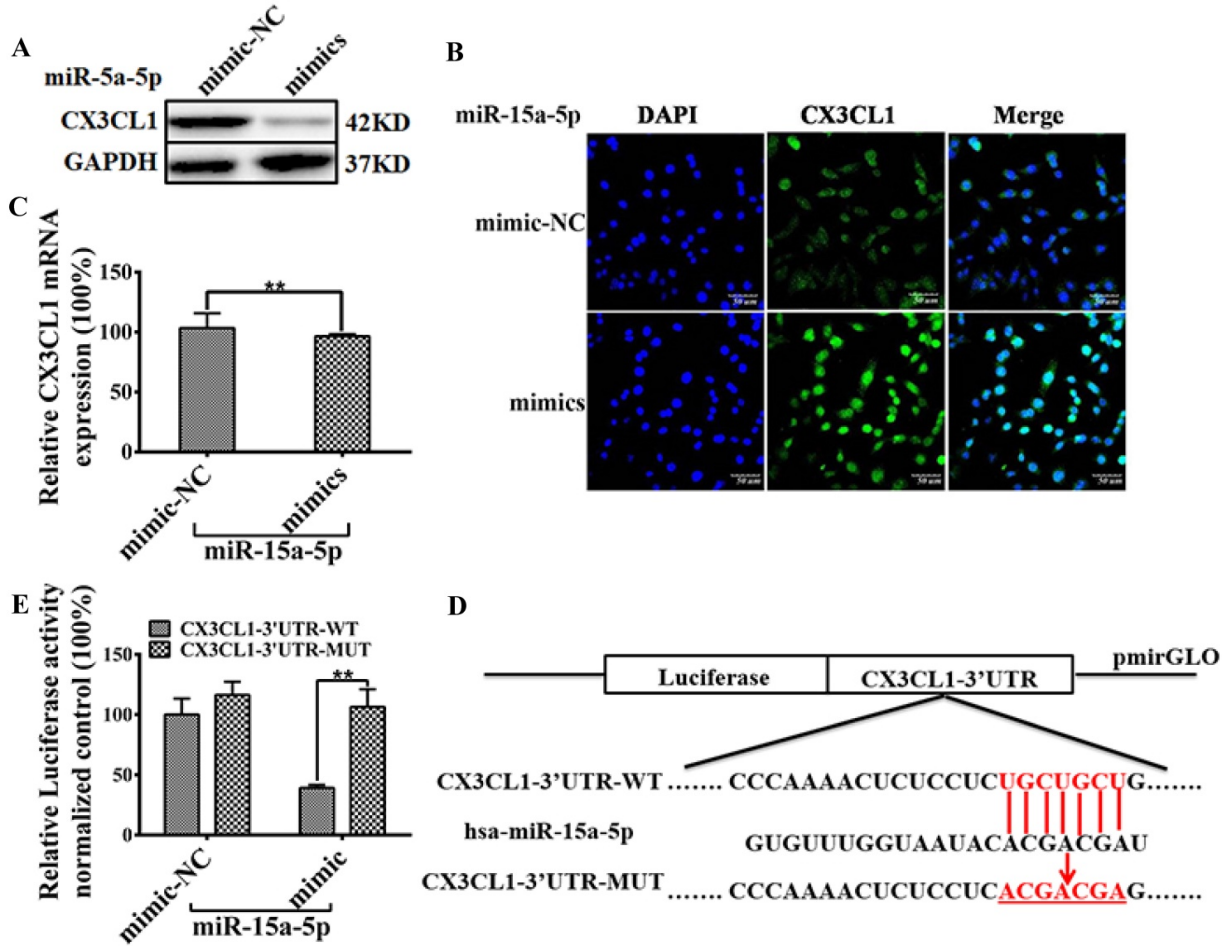
** miR-15a-5p inhibits CX3CL1 protein expression by targeting CX3CL1 3'UTR. A:** The effect of overexpressed miR-15a-5p (mimics) or respective negative control (NC) on the expression of CX3CL1 was examined by western blot analysis in HUVECs. **B:** The representative image shows location of CX3CL1 in HUVECs. The above panels (green) show anti-CX3CL1 antibody reactivity to demonstrate gross morphology. The middle panels (blue) show the DAPI staining for nuclei. The below panels show double immunostained for CX3CL1and nuclei. Scale = 50µm. **C:** Total RNA was isolated and the expression of CX3CL1 was examined by qPCR. GAPDH was used to serve as a loading control. (**P< 0.01). **D:** MiR-15a-5p target sites were predicted in the 3'UTR of CX3CL1 by TargetScan Release 6.2. Bioinformatics analysis unveiled alone miR-15a-5p binding sites on the CX3CL1 3'UTR. The structure of CX3CL1 3'UTR promoter (CX3CL1-UTR-WT) and mutation the hsa-miR-15a-5p binding sites CX3CL1 3'UTR promoter (CX3CL1-UTR-MUT).** E:** HUVECs were respectively transfected with CX3CL1-3'UTR-luc and overexpressed miR-15a-5p (mimics) or respective negative control (NC) 24 hours, then luciferase reporter assays was used to test the transcriptional activity of CX3CL1. Values as the Relative luciferase activity (100%). All experiments were repeated three times in duplicate. (**P< 0.01)

Results of Western blot and immunostaining showed that miR-15a-5p mimics down-regulated the expression level of CX3CL1 in HUVEC (Figure 4A and B). Results of qPCR showed that miR-15a-5p mimics could not regulate the mRNA levels of CX3CL1 in HUVECs (Figure 4C). Bioinformatics analysis showed that there was a miR-15a-5p binding site in CX3CL1 3'UTR (Figure 4E), suggested that CX3CL1 was a potential target for miR-15a-5p. Either the constructs carrying the 3′ UTR of CX3CL1 or the luciferase plasmid vector were co-transfected with miR-15a-5p mimics or N.C into HUVECs. The results showed that the activity of CX3CL1 3'UTR-WT luciferase in co-transfected miR-15a-5p mimics was reduced by 60%, but no such effect was observed in the control group (Figure 4D).

### MiR-15a-5p, CX3CL1 and STAT3 feedback loop regulated the HUVECs proliferation

**Figure 5:**
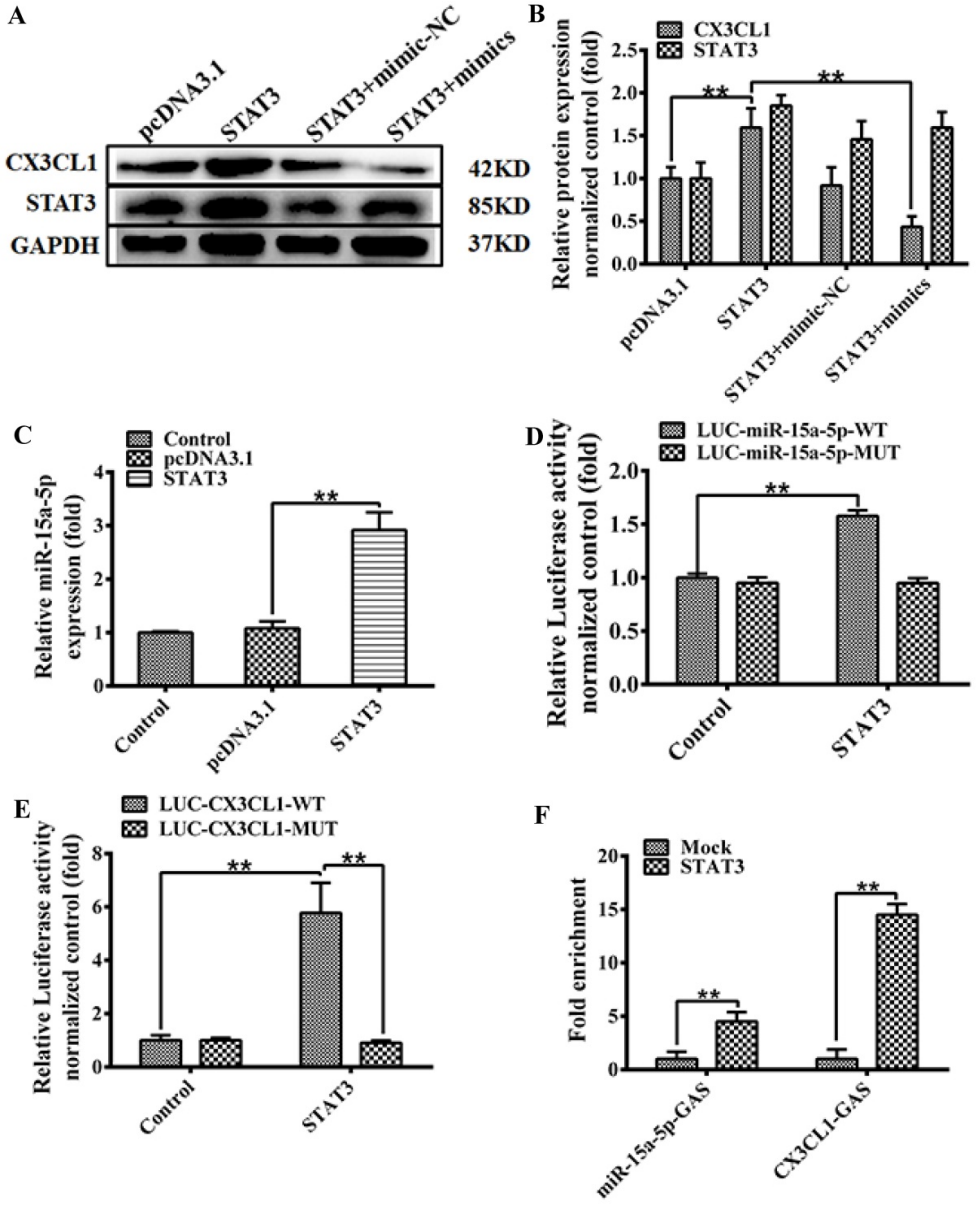
** The model of MiR-15a-5p-CX3CL1 and STAT3 formed a loop to regulate HUVECs proliferation. A and B:** The expression of STAT3 and CX3CL1 expression were analyzed by western blot after 48 hours transfection according to the following groups: pcDNA3.1; pcDNA3.1‐STAT3; pcDNA3.1‐STAT3 + miR‐15a‐5p mimic-NC; pcDNA3.1‐STAT3 + miR‐15a‐5p mimics in HUVECs. GAPDH was a loading control. The data represent mean ± SEM (n = 3). Western blot analysis results in Figure 4B were quantified using the ImageJ software. (**P< 0.01). **C:** HUVECs were transiently transfected with a STAT3, or a control vector (pcDNA3.1) for 48 hours, than the expression of Cx43 was tested by qPCR assays. (**P< 0.01). **D:** HUVECs were transfected with the wildtype miR-15a promoter (LUC-miR-15a-5p-WT), or the miR-15a promoter with mutations in GAS site (LUC-miR-15a-5p-MUT), and transfected with STAT3 48 hours or a control vector (pcDNA3.1). Then the luciferase reporter assays was used to test the transactivity of miR-15a. Values as the Relative luciferase activity (fold). All experiments were repeated three times in duplicate. (**P< 0.01). **E:** HUVECs were transfected with the wild-type CX3CL1 promoter (LUC-CX3CL1-WT), or the CX3CL1 promoter with mutations in GAS site (LUC-CX3CL1-WT), and transfected with STAT3 48 hours or a control vector (pcDNA3.1). Then the luciferase reporter assays was used to test the transactivity of CX3CL1. Values as the Relative luciferase activity (fold). All experiments were repeated three times in duplicate. (**P< 0.01). **F:** HUVECs were transiently transfected with a STAT3, or a control vector (pcDNA3.1) for 48 hours, and ChIP assays were performed by PCR with primers targeting miR-15a-5p as described in “Materials and Methods”. GAPDH show the fold enrichment of STAT3 AT CX3CL1-GAS-sites and miR-15a-5p-GAS-sites at in-gene region in HUVECs. (**P< 0.01)

The expression of STAT3 and CX3CL1 were analyzed by western blot 48 hours after transfection according to the following groups: pcDNA3.1; pcDNA3.1‐STAT3; pcDNA3.1‐STAT3 + miR‐15a‐5p mimic-NC; pcDNA3.1‐STAT3 + miR‐15a‐5p mimics in HUVECs. Western blot results in Figure 5A were quantitatively analyzed with ImageJ software (Figure 5B) (** *P* < 0.01).

We predicted STAT3 targets in CX3CL1 promoter or miR-15a-5p promoter by software analysis. In order to further prove the specificity of binding, we constructed a mutation of STAT3 binding site. The over-expressed STAT3 significantly enhanced the expression of miR-15a-5p (Figure 5C, **P< 0.01). Further studies on the regulation of STAT3 on the patented activity of miR-15a-5p showed that STAT3 significantly increased the transcriptional activity of miR-15a-5p, and mutations in the GAS region of miR-15a-5p promoter showed that the activity of miR-15a-5p promoter mediated by STAT3 returned to the control level (Figure 5D, **P< 0.01). In addition, we validated whether the activity of STAT3-activated CX3CL1 promoter depended on GAS site. We constructed a luciferase reporter vector for CX3CL1 promoter. The results showed that STAT3 could increase the activity of LUC-CX3CL1-WT, and there was no significant difference between the mutant LUC-CX3CL1-MUT activity and the control group (Figure 5E, **P< 0.01). Taken together, these data demonstrate that STAT3 promotes the transcriptional activity of CX3CL1 and miR-15a-5p depending on the GAS element. ChIP-qPCR (chromatin immunoprecipitation) analysis performed to determine whether STAT3 directly binds to CX3CL1 or miR-15a-5p in HUVECs. Immunoprecipitation (IP) was pulled down by the STAT3 antibody in packet processed EA.hy926 cells. As shown in Figure 5F ChIP binding assay STAT3 can bind to the GAS element of the miR-15a-5p and CX3CL1 promoter (Figure 5F).

## Discussion

Studying the pathogenesis of vascular endothelial cell dysfunction and finding therapeutic drugs are potential research directions for atherosclerosis and cardiovascular diseases. Some chemotactic small cytokines, generally 8-12 kDa, are considered as key molecules in the formation and development of atherosclerosis, such as CXCL12, MIF and CCR7 28. CX3CL1, been suggested to be anti-inflammatory and neuroprotective 29, is a potential mediator of both atherosclerosis and metabolic disease 30. We provided evidence that the progressive increase of IL-6 in STAT3 can promote the proliferation of endothelial cells and act as an endogenous promoter of CX3CL1 expression to promote endothelial cell inflammation. Activated STAT3 pathway has been the critical signaling pathway of inflammation and tissue regeneration triggered by almost all pathogenic infections. Many studies have shown that IL-6, a typical proinflammatory cytokine, can participate in acute inflammatory response through STAT3. IL-6 induces the up-regulation of STAT3 in endothelial cells, which is the key factor to activate endothelial dysfunction and induce atherosclerosis. STAT3 is considered to be a key modifier of vascular remodeling and cardiac contractile genes, but its regulation of non-contractile genes is still obscure. Nearly all STATs could recognize the special small DNA palindromic consensus sequence TGCN_2-4_GAA that defines a interferon gamma activation site (GAS) element 31. Our data in this study demonstrate that STAT3 indirectly up-regulates CX3CL1 through its activation on the GAS element.

An ever-growing number of surveys have shown that human vascular endothelial function correlates with miR levels, which play an essential role in the chronic inflammatory process, such as atherosclerosis 32. Emerging pieces of evidence indicate that miRNAs represent a common molecular mechanism that underlies the cell proliferation. There are no studies characterizing miRs that regulate STAT3 signaling responses in inflammation.

MiR-15a-5p is a tumor suppressor, promoting apoptosis and inhibiting cell proliferation by targeting multiple oncogenes, including Bcl-2, Mcl1, CcnD1, and Wnt3A^33^,^34^. MiR-15a down-regulation contributes to myeloma pathogenesis and mediates drug resistance in tumor cells 35,36. However, the functional mechanism of miR-15a in dysfunction of vascular endothelium remains unclear. These results indicate that STAT3 is a potent nuclear factor which promotes the transactivities of miR-15a-5p and CX3CL1 by affecting the formation of the STAT3/GAS complexly both in vitro, in HUVECs. In a word, STAT3 positively modulates expression of CX3CL1 and then down-regulates CX3CL1 inhibits by miR-15a-5p overexpression, thus creating a negative feedback loop to regulate the HUVECs proliferation.

In summary, we implemented data for the initial time that the down-regulation in miR-15a-5p triggers endothelial proliferation and elevates the endothelial inflammation by targeting CX3CL1 and feedback to STAT3 signaling activation. The activity inhibition of CX3CL1 by miR-15a-5p is critical for STAT3 expression and the resultant HUVECs proliferation. These observations reinforce the importance of CX3CL1 in HUVECs proliferation and angiogenesis. Besides, the findings provide chemotaxis insight into the signaling pathways responsible for IL-6-induced STAT3 activation in HUVECs, strongly suggesting the need for cross-talk between CX3CL1 and STAT3 pathway through the bridge-miR-15a-5p. Therefore, the miR-15a-5p and STAT3 in IL-6-induced HUVECs proliferation could provide unique and unexpected therapeutic targets for angiogenesis dependent diseases.

Migration and proliferation of vascular endothelial cells are the basic elements of angiogenesis 37. However, how the miR-15a-5p and nuclear transcription factor STAT3 regulatory pathway network is of great significance to elucidate its different regulatory effects from physiological ones in pathological state. IL-6's effect on STAT3 is time/concentration dependent, and promotes endothelial cell proliferation and chronic inflammation by promoting the expression of CX3CL1. Our research preliminarily analyzed that STAT3/miR-15a-5p has a certain role in maintaining the stability of vascular endothelial function. Under pathological conditions, the unbalanced regulation of CX3CL1 promotes the inflammatory cascade and promotes the formation of atherosclerosis. Regarding the activation of CX3CL1 promoter by STAT3, according to current research, it appears that STAT3 has duality in the activation of gene promoters. It can activate the promoter expression of certain genes, and in some cases can also inhibit the expression of other genes. Subsequent studies need to determine when STAT3 acts on microRNA-15a-5p to maintain angiogenesis and stabilize IL-6's vascular endothelial function, and when STAT3 loses its ability to promote the inflammatory response against microRNA-15a-5p. This path is of great significance for finding potential therapeutic targets for coronary heart disease and revealing the mechanism of coronary atherosclerosis.
